# The Anxiolytic Effect of Aromatherapy on Patients Awaiting Ambulatory Surgery: A Randomized Controlled Trial

**DOI:** 10.1155/2013/927419

**Published:** 2013-12-17

**Authors:** Cheng-Hua Ni, Wen-Hsuan Hou, Ching-Chiu Kao, Ming-Li Chang, Lee-Fen Yu, Chia-Che Wu, Chiehfeng Chen

**Affiliations:** ^1^School of Nursing, College of Nursing, Taipei Medical University, Taipei 11031, Taiwan; ^2^Department of Nursing, Wan Fang Hospital, Taipei Medical University, Taipei 11696, Taiwan; ^3^Head Nurse of Operating Room, Department of Nursing, Wan Fang Hospital, Taipei Medical University, Taipei 11696, Taiwan; ^4^Department of Physical Medicine and Rehabilitation, Taipei Medical University Hospital, Taipei Medical University, Taipei 11031, Taiwan; ^5^School of Gerontology Healthcare Management, College of Nursing, Taipei Medical University, Taipei 11031, Taiwan; ^6^Center for Evidence-Based Medicine, Taipei Medical University, Taipei 11031, Taiwan; ^7^Department of Otolaryngology, Wan Fang Hospital, Taipei Medical University, Taipei 11696, Taiwan; ^8^Department of Otolaryngology, School of Medicine, College of Medicine, Taipei Medical University, Taipei 11031, Taiwan; ^9^Department of Public Health, School of Medicine, College of Medicine, Taipei Medical University, Taipei 11031, Taiwan; ^10^Division of Plastic Surgery, Department of Surgery, Wan Fang Hospital, Taipei Medical University, Taipei 11696, Taiwan; ^11^Evidence-Based Medicine Center, Wan Fang Hospital, Taipei Medical University, Taipei 11696, Taiwan

## Abstract

The aim of this study was to determine if aromatherapy could reduce preoperative anxiety in ambulatory surgery patients. A total of 109 preoperative patients were randomly assigned to experimental (bergamot essential oil) and control (water vapor) conditions and their responses to the State Trait Anxiety Inventory and vital signs were monitored. Patients were stratified by previous surgical experience, but that did not influence the results. All those exposed to bergamot essential oil aromatherapy showed a greater reduction in preoperative anxiety than those in the control groups. Aromatherapy may be a useful part of a holistic approach to reducing preoperative anxiety before ambulatory surgery.

## 1. Introduction

Aromatherapy is a form of complementary and alternative medicine that uses essential oils to affect a patient's mood and health. In addition to music, relaxation, guided imagery, and massage, aromatherapy has been used by nurses as part of a holistic approach to minimize preoperative anxiety [1]. Heightened patient anxiety may cause increased difficulty in the procedure, increased physical discomfort, and the subsequent need for higher doses of medication for procedural sedation and postoperative pain control [2].

Several studies have used aromatherapy as an adjunct to different procedures and the results have been mixed. For example, there was a reduction in preoperative anxiety in general surgical patients [3, 4] and those with postoperative nausea [5] as well as school teachers with generalized anxiety [6] and nursing home residents [7]. Aromatherapy was no better than placebo in reducing anxiety prior to endoscopy [8, 9], stem cell infusion [10], radiotherapy [11], or therapeutic abortion [12].

Many common plant oils are commonly utilized as anxiolytics. These include lavender (*Lavandula angustifolia*), rose (*Rosa damascena*), bergamot (*Citrus aurantium*,subsp *bergamia*), and sandalwood (*Santalum album*) [13]. Bergamot has been utilized in a number of clinical studies (e.g., [6, 10–12]) and was selected for use in this one.

The preoperative waiting period for ambulatory surgery is usually briefer than that for general surgery, and it is important to have a quick and convenient mechanism to control anxiety. The aim of this study was to determine if aromatherapy with bergamot essential oil could reduce preoperative anxiety in patients awaiting ambulatory surgery.

## 2. Methods

### 2.1. Participants

A total of 116 patients who were admitted to Taipei Medical University, Municipal Wan Fang Hospital, for ambulatory surgery from May 1st to September 30th, 2012, were selected by utilization of a random number table. The investigator contacted the patients by phone one day prior to surgery and invited their participation in a study where they would be asked to complete questionnaires about anxiety. Inclusion criteria were age between 18 and 65, clear consciousness, and the ability to communicate with researchers. Patients were excluded if they showed evidence of mental illness, used sedatives before surgery, were scheduled for a nasal procedure or major or high risk surgery, could not read and write Chinese, or the waiting time before the expected surgery was less than 45 minutes. Seven patients declined to participate. The remaining 109 patients signed informed consent on the day of surgery. The study was approved by the Institutional Review Board of Municipal Wan Fang Hospital.

### 2.2. Procedure

As part of the registration, patients completed the State Trait Anxiety Inventory (STAI) and their vital signs were recorded. In the preparation room, they were exposed to the experimental (bergamot essential oil aromatherapy) or control (water vapor) condition for thirty minutes and they completed the STAI a second time and vital signs were again recorded. They then proceeded to surgery.

### 2.3. Instruments

The device for aromatherapy, an ultrasonic aroma diffuser (MUJI, Tokyo, Japan), was hidden behind a screen on the ward and reset before use with each patient. Bergamot essential oil was purchased from Oshadhi (AYUS Ätherische Öle und Naturrohstoffe GmbH, Bühl, Germany).

The STAI has been used extensively since 1966 [14]. It consists of 20 statements (e.g., “I am tense,” “I am relaxed”) and respondents rate the intensity of their feelings about each at that moment from 1 (not at all) to 4 (very much so) so that scores range from 20 to 80, higher scores indicating greater anxiety (low anxiety: 20–39; moderate anxiety: 40–59; and high anxiety: 60–80). The validated Chinese version [15] was used in this study. The STAI takes fewer than five minutes to complete and can be scored quickly.

### 2.4. Statistical Analysis

Continuous data were presented as mean ± standard deviation (SD), but the changes in STAI, heart rate, and blood pressure were presented as median and interquartile range (IQR) due to their nonnormal distribution. The changes in STAI, heart rate, and blood pressure between the two groups were assessed with the nonparametric Mann-Whitney test and other continuous data between groups with the two independent samples *t*-test. The changes from baseline STAI, heart rate, and blood pressure within groups were evaluated with the nonparametric Wilcoxon signed ranks test. Categorical data were shown by number and percentage, and their associations with treatment groups were evaluated by Fisher's exact test. Statistical assessments were two-tailed and considered significant at *P* < 0.05. Statistical analyses were performed using SPSS 15.0 statistics software (SPSS Inc, Chicago, IL, USA).

## 3. Results

**Figure 1:**
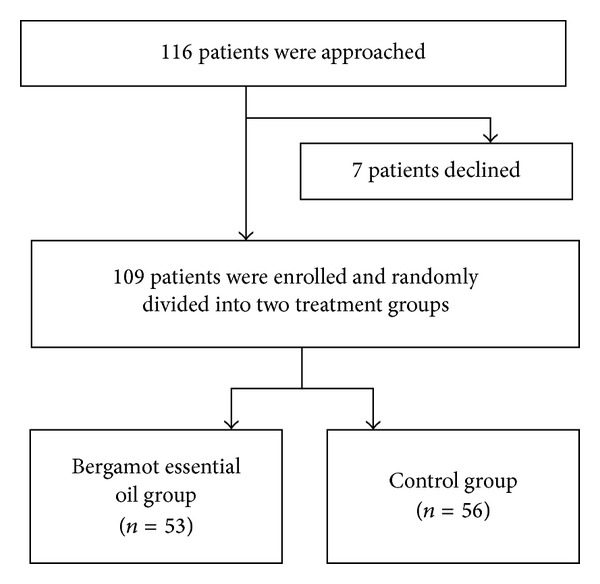
Patient flow chart.

Again, with the use of a random number table, the 109 subjects were randomly assigned into two groups: bergamot essential oil (*n* = 53) and control (*n* = 56) (Figure 1). Most of the baseline characteristics were comparable between the two groups (*P* > 0.05) except for surgical experience. Significantly more patients with surgical experience were randomly assigned to the control group than to the bergamot essential oil group (80.4% versus 56.6%, *P* = 0.013). Since two treatment groups were not comparable, the associations between anxiety scores and vital sign parameters and aromatherapy were stratified by surgical experience based on the assumption that such prior experience might influence the treatment effect (Table 1).

The 34 patients without surgical experience included 23 in the bergamot essential oil group and 11 in the control group. The STAI scores decreased more in the bergamot group than in the control group (−3.0 versus −2.0, *P* = 0.021). There were no significant differences between groups in changes in heart rate or blood pressure. The STAI score, heart rate, and blood pressure in the bergamot group significantly decreased from baseline, as the median changes in STAI, heart rate, systolic blood pressure (SBP), and diastolic blood pressure (DBP) were −3.0 (*P* < 0.001), −6.0 beats/min (*P* = 0.015), −11.0 mmHg (*P* < 0.001), and −5.0 mmHg (*P* = 0.012), respectively. No significant changes in STAI, heart rate, SBP, or DBP were observed in the control group (*P* > 0.05).

The 75 patients with surgical experience included 30 in the bergamot essential oil and 45 in the control group. The STAI score decreased more in the bergamot group than in the control group (−4.0 versus −1.0, *P* = 0.005). There was no significant difference between groups in changes in heart rate or blood pressure. The STAI score, heart rate, and SBP in the bergamot group significantly decreased when compared to baseline, as the median changes in STAI, heart rate, and SBP were −4.0 (*P* < 0.001), −3.5 beats/min (*P* = 0.004), and −11.0 mmHg (*P* < 0.001), respectively. Similar results were observed in the control group as the median changes in STAI, heart rate, and SBP were −1.0 (*P* = 0.019), −5.0 beats/min (*P* < 0.001), and −7.0 mmHg (*P* < 0.001), respectively (Table 2).

In addition, no significant difference in baseline STAI was observed between those with and without surgical experience (median: 44.0 versus 45.0, *P* = 0.385). There were also no significant differences in post-treatment STAI or STAI change between those with and without surgical experience. No negative effects were reported by either group.

## 4. Discussion

Patients with surgical experience in both the experimental and control groups showed improvement in anxiety scores and vital signs, but the decrease in anxiety was significantly greater in the aromatherapy group. Patients without surgical experience, regardless of the group, also showed improvement in STAI scores, heart rate, and SBP, but again, the decrease in anxiety was more in the aromatherapy group. Although the findings were robust, the analysis would have been stronger if the randomization had been stratified for prior surgical experience, and this is a limitation of the study.

The mechanism of action of bergamot is not completely understood but may be due to the exocytic and carrier-mediated release of discrete amino acids action as neurotransmitters that interfere with normal and pathological synaptic plasticity [16]. In an animal study, bergamot attenuated hypothalamic-pituitary-adrenal activity by reducing the corticosterone response to stress [17].

Aromatherapy, then, has an important role to play in holistic nursing practice, especially in time-limited situations such as the preoperative wait before ambulatory surgery; however, as demonstrated by improvement in the control group, nursing care itself helps to alleviate preoperative anxiety. Caring therapeutic use of the self as well as holistic interventions such as hypnosis, guided imagery, music, touch, and aromatherapy is essential when dealing with this common and distressing problem for most surgical patients [1].

## 5. Conclusions

Regardless of previous surgical experience, patients exposed to bergamot essential oil aromatherapy were less anxious than controls. Aromatherapy may be a useful part of a holistic approach to reducing preoperative anxiety before ambulatory surgery.

## Figures and Tables

**Table 1:** Summary of baseline characteristics of the study population (*n* = 109).

	Total (*n* = 109)	Bergamot essential oil (*n* = 53)	Control (*n* = 56)	*P* value
Age^1^ (years)	45.4 (11.7)	45.0 (12.2)	45.9 (11.4)	0.687
Gender^2^				
Male	44 (40.4%)	20 (37.7%)	24 (42.9%)	0.697
Female	65 (59.6%)	33 (62.3%)	32 (57.1%)	
BMI^1^ (kg/m^2^)	23.7 (3.9)	23.8 (4.5)	23.6 (3.2)	0.712
Education^2^				
Elementary school or below	14 (12.8%)	5 (9.4%)	9 (16.1%)	0.238
High school	37 (33.9%)	22 (41.5%)	15 (26.8%)	
College or above	58 (53.2%)	26 (49.1%)	32 (57.1%)	
Surgery^2^				
Neurosurgery	53 (48.6%)	26 (49.1%)	27 (48.2%)	0.791
Plastic surgery	29 (26.6%)	12 (22.6%)	17 (30.4%)	
General surgery	13 (11.9%)	6 (11.3%)	7 (12.5%)	
ENT	4 (3.7%)	2 (3.8%)	2 (3.6%)	
Urology	3 (2.8%)	2 (3.8%)	1 (1.8%)	
Obstetrics and gynaecology	3 (2.8%)	3 (5.7%)	0 (0.0%)	
Breast surgery	2 (1.8%)	1 (1.9%)	1 (1.8%)	
Dermatology	2 (1.8%)	1 (1.9%)	1 (1.8%)	
With experience of surgery^2^				
Yes	75 (68.8%)	30 (56.6%)	45 (80.4%)	0.013*
No	34 (31.2%)	23 (43.4%)	11 (19.6%)	
Smoking^2^				
Yes	31 (28.4%)	15 (28.3%)	16 (28.6%)	1.000
No	78 (71.6%)	38 (71.7%)	40 (71.4%)	
Drinking^2^				
Yes	36 (33.0%)	16 (30.2%)	20 (35.7%)	0.550
No	73 (67.0%)	37 (69.8%)	36 (64.3%)	
Heart rate^1^ (beat/min)	77.2 (10.2)	77.1 (10.0)	77.3 (10.5)	0.901
SBP^1^ (mmHg)	127.7 (17.3)	129.3 (15.9)	126.2 (18.5)	0.354
DBP^1^ (mmHg)	76.0 (11.1)	76.7 (11.3)	75.4 (11.0)	0.531
Baseline STAI^1^	43.6 (11.0)	45.4 (10.8)	42.0 (11.0)	0.106

*P < 0.05 indicates a significant difference between groups.

^1^Data are presented as mean and SD.

^2^Data are presented as number and percent.

**Table 2:** Summary of the mean changes in anxiety scores and vital sign parameters with stratification by surgical experience.

Mean change from baseline	Bergamot essential oil	Control	*P* value
Without experience of surgery (*n* = 34)			
STAI change	−3.0 (−10.0, −2.0)^†^	−2.0 (−3.0, 1.0)	0.021*
Heart rate change	−6.0 (−9.0, 1.0)^†^	−5.0 (−10.0, 2.0)	0.877
SBP change	−11.0 (−16.0, −8.0)^†^	−8.0 (−11.0, 4.0)	0.051
DBP change	−5.0 (−9.0, 0.0)^†^	1.0 (−8.0, 6.0)	0.140
With experience of surgery (*n* = 75)			
STAI change	−4.0 (−8.0, −1.0)^†^	−1.0 (−4.0, 1.0)^†^	0.005*
Heart rate change	−3.5 (−5.0, 0.0)^†^	−5.0 (−10.0, −1.0)^†^	0.084
SBP change	−11.0 (−17.0, −2.0)^†^	−7.0 (−12.0, −1.0)^†^	0.280
DBP change	−1.0 (−6.0, 2.0)	−4.0 (−6.0, 5.0)	0.649

Data are presented as median and IQR. *P < 0.05 indicates a significant difference between groups; ^†^indicates a significant change from baseline.
